# The Krüppel-like factor 2 and Krüppel-like factor 4 genes interact to maintain endothelial integrity in mouse embryonic vasculogenesis

**DOI:** 10.1186/1471-213X-13-40

**Published:** 2013-11-22

**Authors:** Aditi R Chiplunkar, Benjamin C Curtis, Gabriel L Eades, Megan S Kane, Sean J Fox, Jack L Haar, Joyce A Lloyd

**Affiliations:** 1 Department of Human and Molecular Genetics, Virginia Commonwealth University, Richmond, Virginia 23298-0035, USA; 2 Department of Anatomy and Neurobiology, Virginia Commonwealth University, Richmond, Virginia 23298-0709, USA; 3 Massey Cancer Center, Virginia Commonwealth University, Richmond, Virginia 23298-0035, USA

**Keywords:** KLF2, KLF4, Gene interactions, Embryonic vascular integrity, Endothelial cell development

## Abstract

**Background:**

Krüppel-like Factor 2 (KLF2) plays an important role in vessel maturation during embryonic development. In adult mice, KLF2 regulates expression of the tight junction protein occludin, which may allow KLF2 to maintain vascular integrity. Adult tamoxifen-inducible Krüppel-like Factor 4 (KLF4) knockout mice have thickened arterial intima following vascular injury. The role of KLF4, and the possible overlapping functions of KLF2 and KLF4, in the developing vasculature are not well-studied.

**Results:**

Endothelial breaks are observed in a major vessel, the primary head vein (PHV), in KLF2-/-KLF4-/- embryos at E9.5. KLF2-/-KLF4-/- embryos die by E10.5, which is earlier than either single knockout. Gross hemorrhaging of multiple vessels may be the cause of death. E9.5 KLF2-/-KLF4+/- embryos do not exhibit gross hemorrhaging, but cross-sections display disruptions of the endothelial cell layer of the PHV, and these embryos generally also die by E10.5. Electron micrographs confirm that there are gaps in the PHV endothelial layer in E9.5 KLF2-/-KLF4-/- embryos, and show that the endothelial cells are abnormally bulbous compared to KLF2-/- and wild-type (WT). The amount of endothelial Nitric Oxide Synthase (eNOS) mRNA, which encodes an endothelial regulator, is reduced by 10-fold in E9.5 KLF2-/-KLF4-/- compared to KLF2-/- and WT embryos. VEGFR2, an eNOS inducer, and occludin, a tight junction protein, gene expression are also reduced in E9.5 KLF2-/-KLF4-/- compared to KLF2-/- and WT embryos.

**Conclusions:**

This study begins to define the roles of KLF2 and KLF4 in the embryonic development of blood vessels. It indicates that the two genes interact to maintain an intact endothelial layer. KLF2 and KLF4 positively regulate the eNOS, VEGFR2 and occludin genes. Down-regulation of these genes in KLF2-/-KLF4-/- embryos may result in the observed loss of vascular integrity.

## Background

Vasculogenesis is a dynamic process in mammalian embryonic development. Endothelial precursors, known as angioblasts, initiate intraembryonic vascular development by murine embryonic day 7.0 (E7.0) forming a primary capillary plexus (Reviewed in) [1]. During maturation, endothelial cells recruit mesenchymal cells or vascular smooth muscle cell (VSMC) progenitors to the surface of the vessels, and these cells organize into layers around the tube to support it (Reviewed in) [2]. By E7.5, endothelial cells also promote and maintain VSMC differentiation [3].

Krüppel-like Factor 2 (KLF2) is a zinc finger DNA binding protein with essential roles in vascular endothelial biology. The gene is expressed in endothelial cells in the developing mouse as early as E8.5 [4]. KLF2 knockout mice die *in utero* between E10.5 and E14.5, and the time of death is dependent on the genetic background [4-7]. Angiogenesis and vasculogenesis appear grossly normal in viable E11.5 KLF2-/- mice. Kuo et al. generated KLF2 knockout embryos, and concluded that death is due to hemorrhaging and a lack of integrity in the smooth muscle layers that surround vessels from around E11.5 [5]. Our previous studies show that in the absence of KLF2, the dorsal aortae are abnormal in E10.5 FVB/N mice. The endothelial cell layer lacks integrity and there are erythroid cells outside of the aortae [7]. In another study, Wu et al. showed that KLF2-/- embryos have normal endothelial cell development, but a failure of mural cells to migrate around endothelial cells to stabilize blood vessels [8]. Recent findings suggest that KLF2 plays an important role in endothelial barrier function in adult mice. It positively regulates expression of the tight junction protein occludin and modification of myosin light chain that is important for the integrity of the endothelial layer and to avoid vascular leakage [9].

Krüppel-like Factor 4 (KLF4) is a member of the Krüppel-like transcription factor family, and is ~90% similar to KLF2 in its zinc finger DNA binding domain, suggesting the factors could have common target sequences. KLF4 is expressed in mesenchymal tissue, endothelium and epithelium by E10.5 [10], and is essential for skin barrier function during development [11]. KLF4 knockout mice die soon after birth [10]. In tamoxifen-inducible KLF4-/- adult mice, vascular injury-induced repression of smooth muscle cell markers is delayed, thus indicating that KLF4 controls phenotypic switching of vascular smooth muscle cells [12-16]. Vascular abnormalities have not been reported during the early embryonic development of KLF4-/- mice.

In tissue culture, KLF2 plays a role as a molecular transducer of fluid shear forces, thus directly or indirectly regulating a number of endothelial genes including endothelial Nitric Oxide Synthase (eNOS) and endothelin [17]. KLF4 is induced by laminar shear stress in human umbilical vein endothelial cells (HUVECs) and transactivates the eNOS and thrombomodulin (TM) promoters. TM and eNOS are important in vascular tone regulation and maintenance of intact endothelium [18].

KLF2 and KLF4 are induced by shear stress and activated by the MEK5/MEF2 signaling pathway. Using genome wide transcriptional profiling of HUVEC cells overexpressing KLF2, KLF4 or constitutively active MEK5, it was shown that ~60% of the genes activated by MEK5 were also regulated by either KLF2 or KLF4. These studies suggest that there is mechanistic and functional conservation between KLF2 and KLF4 in vascular endothelial cells [18].

The combined roles of KLF2 and KLF4 have thus far been studied only in *in vitro* endothelial models. Because the two factors are evolutionarily closely related, it was of interest to determine if they had overlapping roles in embryonic development. This study shows for the first time that there are interactions between the KLF2 and KLF4 genes during vascular development.

## Results

### KLF2-/-KLF4-/- embryos show hemorrhaging in the cranial region

KLF2-/- embryos die between E10.5 and E14.5 [6,7] and KLF4-/- mice die perinatally [11]. Of the 67 embryos obtained from nine KLF2+/-KLF4+/- matings, at least 4 E10.5 KLF2-/-KLF4-/- embryos were expected to be obtained. However, no live KLF2-/-KLF4-/- embryos were observed at E10.5, and the necrotic KLF2-/-KLF4-/- embryos that were recovered lacked integrity, and could not successfully be processed for sectioning. Nevertheless, in these E10.5 KLF2-/-KLF4-/- embryos, gross hemorrhaging was evident in the head and trunk (data not shown), suggesting that this might be a contributing factor in their early death. Surprisingly, of the 7 KLF2-/-KLF4+/- embryos obtained, only one embryo had a beating heart and the other 6 were not viable. Gross hemorrhaging was also observed in a majority of these embryos. At E10.5, KLF2-/- embryos were viable as evidenced by a beating heart, and did not exhibit gross cranial hemorrhaging. This thus suggests that the additional loss of one KLF4 allele from KLF2-/- embryos results in the hemorrhaging phenotype, implying an interaction between the KLF2 and KLF4 genes.

**Figure 1:**
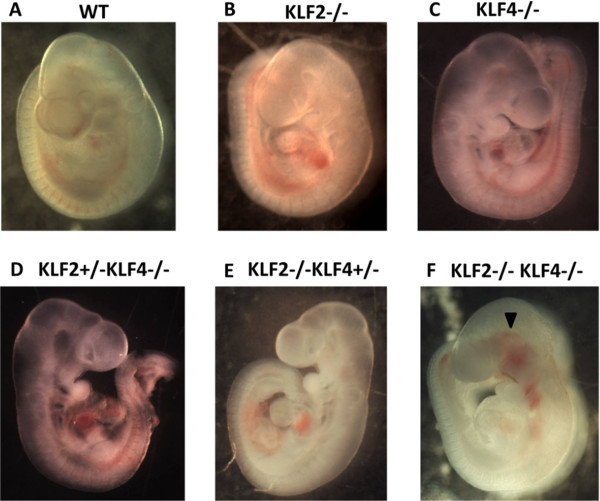
**Ablation of KLF2 and KLF4 leads to cranial hemorrhaging by E9.5.** Light micrographs of E9.5 mouse embryo whole mounts were taken at 16X magnification. **A)** WT (n = 4); **B)** KLF2-/- (n = 3); **C)** KLF4-/- (n = 3); **D)** KLF2+/-KLF4-/- (n = 3); **E)** KLF2-/-KLF4+/- (n = 4) embryos are grossly normal. **F)** KLF2-/-KLF4-/- (n = 3) embryos show hemorrhaging and/or enlarged blood vessels in the head region. Arrowhead indicates hemorrhaging.

To further investigate the vascular phenotype of KLF2-/-KLF4-/- compared to KLF2 and KLF4 single knockout embryos, macroscopic examination of intact embryos and light microscopic examination of tissue sections was performed to assess morphological abnormalities. E9.5 embryos were collected from KLF2+/-KLF4+/- matings. The expected numbers of all genotypes were obtained, yielding WT (n = 4), KLF2+/-KLF4+/- (n = 7), KLF2-/- (n = 3), KLF4-/- (n = 3), KLF2-/-KLF4+/- (n = 4), KLF2+/-KLF4-/- (n = 3), and KLF2-/-KLF4-/- (n = 3) embryos. All of the E9.5 KLF2-/-KLF4-/- embryos were viable, as evidenced by a heartbeat. Cranial hemorrhaging was not observed in the WT (Figure 1A), KLF2-/- (Figure 1B) or KLF4-/- (Figure 1C) embryos. KLF2-/-KLF4+/- and KLF2+/-KLF4-/- embryos also did not show gross hemorrhaging (Figure 1D and E). One of the KLF2-/-KLF4-/- embryos showed gross hemorrhaging macroscopically, in the cranial region, beginning rostrally at the level of optic vesicle and ending caudally at the level of the first branchial arch (Figure 1F). Gross and microscopic examination confirmed that hemorrhaging in E9.5 KLF2-/-KLF4-/- embryos is confined to the primary head vein (PHV) and the rostral portion of the anterior cardinal vein, into which the PHV drains. Bleeding penetrates at some levels, all the way to the midline infiltrating the supporting cranial mesenchyme, a precursor tissue to vascular smooth muscle. Gross hemorrhaging in KLF2-/-KLF4-/- embryos is more severe and occurs in vessels other than the PHV by E10.5, consistent with the time of death.

**Figure 2:**
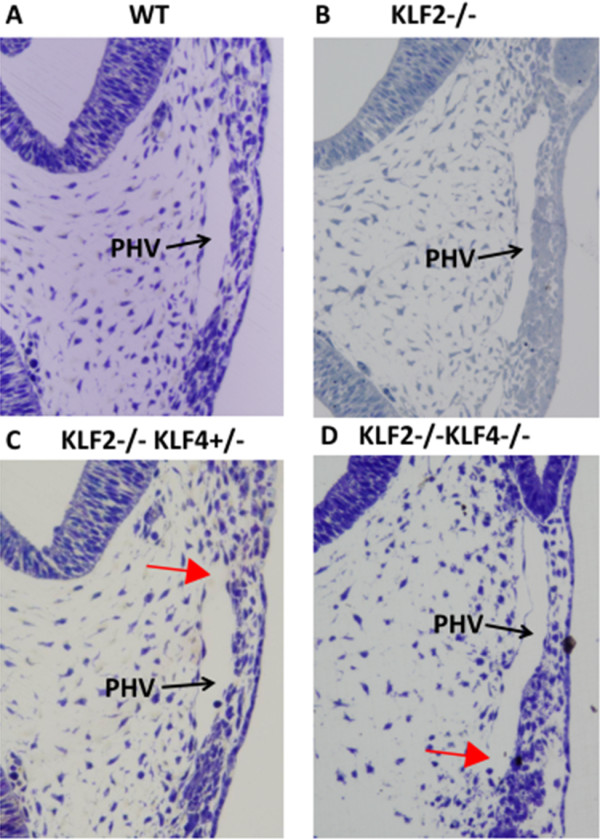
**KLF2-/-KLF4-/- primary head vein lacks continuous endothelial layer at E9.5.** Light micrographs of 6 μm sections were taken at 200X magnification. **A)** WT and **B)** KLF2-/- (n = 4) shows normal looking primary head vein; **C)** Two of the four KLF2-/-KLF4+/- embryos have gaps in the endothelial layer of the primary head vein (n = 4); **D)** KLF2-/-KLF4-/- primary head vein lacks a continuous endothelial layer (n = 3). Red arrows indicate apparent gaps in the endothelial layer. Black arrows point at the lumen of the Primary Head Vein (PHV).

Tissue-sections of the PHV at the level of the optic vesicle reveal a continuous endothelium in E9.5 WT (Figure 2A) and KLF2-/- (Figure 2B) embryos. In KLF2-/-KLF4-/- embryos, there are apparent gaps between adjoining endothelial cells (Figure 2D). Endothelial disruption of the PHV, which was not evident at the gross level, is also seen at the microscopic level in two of the four KLF2-/-KLF4+/- embryos that were examined (Figure 2C). This phenotype is variable, and the other two E9.5 KLF2-/-KLF4+/- embryos appeared like wild-type, having no PHV phenotype. The presence of apparent gaps in the endothelial layer suggests a lack of vascular integrity. No abnormal phenotype was seen in KLF2+/-KLF4-/- embryos (data not shown), suggesting that complete KLF2 ablation is required for this abnormal vascular phenotype. The fact that the KLF2-/-KLF4-/- and KLF2-/-KLF4+/- phenotypes are more severe than KLF2-/- indicates that KLF4 plays a larger role in vascular development than previously recognized and is complemented by KLF2. Serial tissue sections of entire E9.5 embryos were examined for this study, and the abnormal vascular phenotype in KLF2-/-KLF4-/- and some KLF2-/-KLF4+/- embryos is observed only in the primary head vein. The primary head vein is one of the major blood vessels at this stage of development. Although the PHV is the only vessel to exhibit hemorrhaging at E9.5, based on the gross hemorrhaging observed by E10.5, other vessels in KLF2-/-KLF4-/- embryos lack integrity by this later time point.

### Electron micrographs confirm disruption of the endothelial layer of the primary head vein in KLF2-/-KLF4-/- embryos at E9.5

**Figure 3:**
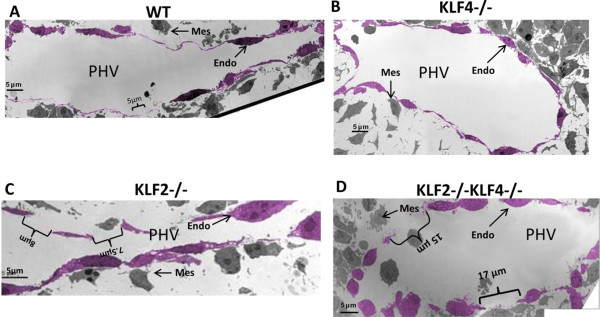
**Electron micrographs confirm disruption of the endothelial layer of the primary head vein in E9.5 KLF2-/-KLF4-/- embryos.** Images were taken of the PHV at the level of the optic vesicle. The endothelial cell layer was pseudo-colored using GIMP (GNU Image Manipulation Program) version 2.6 open-source digital photo editing software. **A)** WT embryo (n = 1) has a continuous endothelial layer with slight gaps less than 5 μm in length. **B)** Only slight gaps were observed in KLF4-/- embryo (n = 1). **C)** One of the two KLF2-/- embryos presented a few gaps of 8 μm and 7.5 μm in length. **D)** KLF2-/-KLF4-/- embryos (n = 2) had consistent disruptions of the endothelial membrane with gaps as long as 17 μm. PHV: primary head vein lumen: Endo: endothelial cell; Mes: mesenchymal cell.

The apparent endothelial disruption at the primary head vein observed in E9.5 KLF2-/-KLF4-/- embryos was more completely characterized using transmission electron microscopy (TEM). Electron micrographs of the PHV at the level of the optic vesicle are shown in Figure 3. In the WT, the endothelium is continuous with gaps ≤ 5 μm (Figure 3A). The typical flattened spindle shape of the endothelial cells seen at the light level is confirmed. The KLF4-/- embryo also has endothelium that is generally continuous with gaps that are indistinguishable from WT (Figure 3B). KLF2-/- embryos have a modest and variable phenotype. One KLF2-/- PHV has no visible abnormalities of the endothelium, with gaps appearing identical to the WT and KLF4-/- embryos (data not shown). Another KLF2-/- shows multiple ≤ 8 μm gaps along the medial aspect of the vessel (Figure 3C). Endothelial cells are present and outline the lumen of the PHV. KLF2-/-KLF4-/- embryos have endothelial disruption and more frequent gaps ≤ 20 μm on both the medial and lateral aspects of the fragmented vessel wall (Figure 3D). Furthermore, the endothelial cells in the KLF2-/-KLF4-/- vessels are more bulbous in shape compared to KLF2-/-, KLF4-/- and WT. These findings confirm the disruption of the endothelial layer of the PHV observed at the light microscopy level in E9.5 KLF2-/-KLF4-/- embryos. In all four genotypes tested, mesenchymal cells are found near the PHV at E9.5, but muscle cells have not yet formed a continuous layer surrounding the vessel (Figure 3A-3D).

### KLF2 and KLF4 regulate eNOS mRNA expression at E9.5

To begin to elucidate the molecular mechanism by which KLF2 and KLF4 ablation results in endothelial disruption, the amounts of mRNA for endothelial cell regulators were quantified. Genes were selected for their established role in vascular development, a similar KO phenotype to KLF2-/-KLF4-/-, the presence of potential KLF2 and KLF4 binding sites (CCRCCC) in the proximal promoter [19,20], and a high ratio of expression in endothelial compared to other cell types at E9.5.

**Figure 4:**
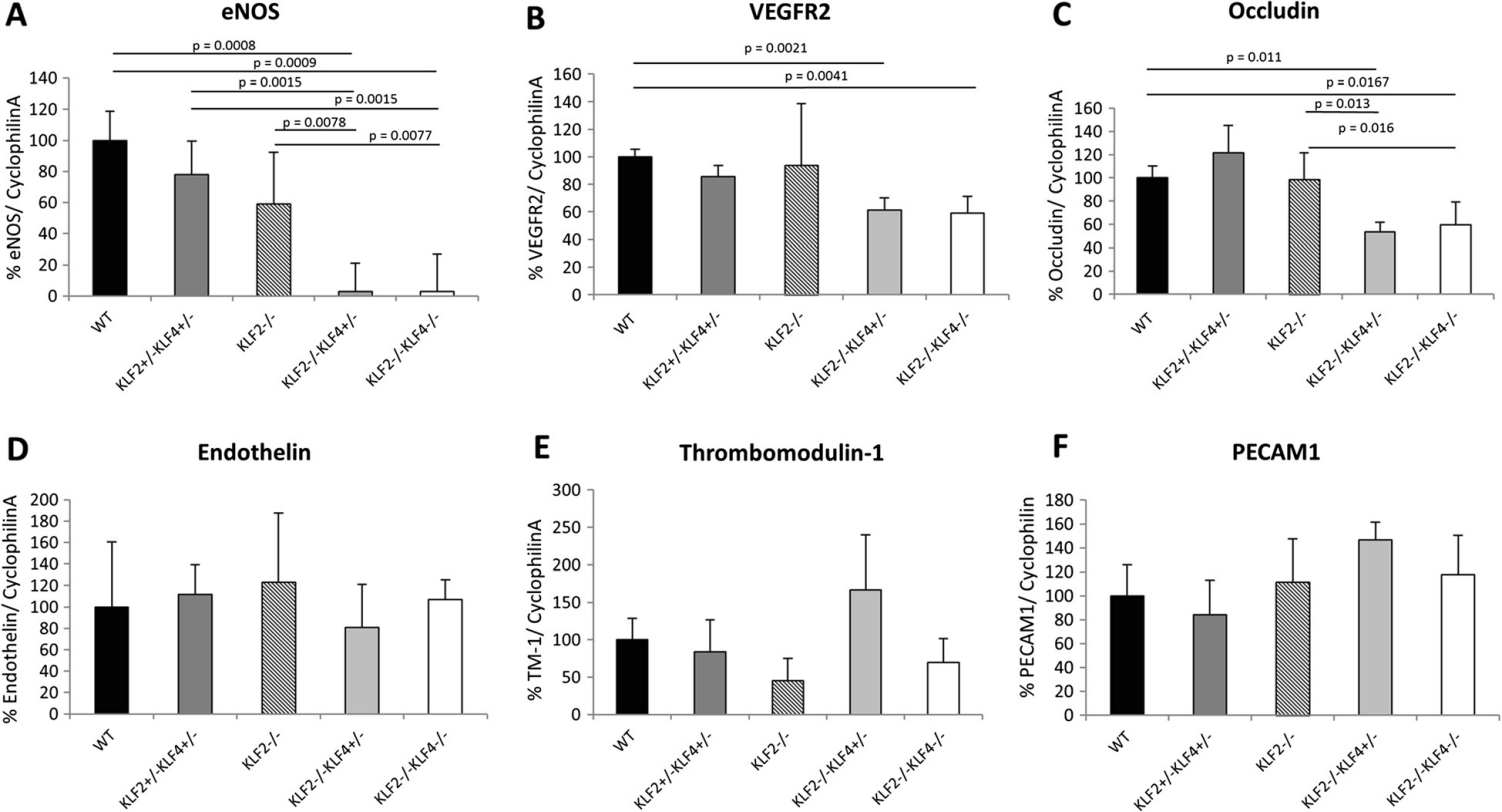
**KLF2 and KLF4 gene ablation decreases eNOS mRNA expression in E9.5 embryos. A)** qRT-PCR revealed that eNOS mRNA was reduced in E9.5 KLF2-/-KLF4+/- and KLF2-/-KLF4-/- embryos compared to KLF2-/- (p = 0.0078 and p = 0.0077, respectively) and WT (p = 0.0008 and p = 0.0009, respectively) embryos of the same age. **B)** VEGFR2 mRNA is significantly reduced in KLF2-/-KLF4+/- and KLF2-/-KLF4-/- compared to WT (p = 0.0021 and p = 0.0041, respectively) but not KLF2-/-. **C)** Occludin mRNA is also significantly reduced in KLF2-/-KLF4+/- and KLF2-/-KLF4-/- compared to WT (p = 0.0167 and p = 0.011, respectively) and KLF2-/- (p = 0.013 and p = 0.016, respectively). **D)** Endothelin, **E)** Thrombomodulin-1 and **F)** PECAM1 mRNA amounts are not significantly different in KLF2-/-KLF4-/- and KLF2-/-KLF4+/- compared to WT and single knockout embryos. n = 3 for each genotype.

Expression of eNOS mRNA in E9.5 mouse embryos was quantified and normalized to the ubiquitously-expressed housekeeping gene, Cyclophilin A (Figure 4). While no difference in eNOS mRNA expression is noted between WT and KLF2-/-, eNOS mRNA expression in KLF2-/-KLF4-/- is significantly lower than in WT (p = 0.0009), KLF2+/-KLF4+/- (p = 0.0015) and KLF2-/- (p = 0.0077) embryos using a two-tailed student t-test. Additionally, using the same statistical test, eNOS expression in KLF2-/-KLF4+/- is significantly lower than in WT (p = 0.0008), KLF2+/-KLF4+/- (p = 0.0015) and KLF2-/- embryos (p = 0.0078) (Figure 4A). Unfortunately, KLF4-/- embryos were not obtained for the gene expression studies; we cannot rule out the possibility that KLF4 knockout alone affects endothelial cell gene regulation. The data indicate that both KLF2 and KLF4 are required for eNOS regulation. Interestingly, lower eNOS mRNA expression correlates with the more severe phenotype in KLF2-/-KLF4+/- and KLF2-/-KLF4-/- embryos compared to the other genotypes. KLF2 and KLF4 positively affect the expression of eNOS, which is a regulator of endothelial homeostasis and vasculogenesis. eNOS-/- mice are not embryonic lethal and their phenotype is indistinguishable from eNOS+/- and wild-type mice in general appearance and histology [21]. Thus, other genes downstream of KLF2 and KLF4 must also contribute to the embryonic lethality of KLF2-/-KLF4-/-.

VEGFR2 (Figure 4B) and occludin (Figure 4C) mRNA expression is significantly reduced in KLF2-/-KLF4-/- and KLF2-/-KLF4+/- embryos compared to WT. The decrease in these mRNAs is modest (2-fold), however VEGFR2 and occludin are not endothelial cell-specific, and their reduction in endothelial cells may be more pronounced. VEGFR2 induces eNOS expression [22] and occludin is a tight junction protein [23]. Therefore, the reduced expression of these genes could logically contribute to the observed vascular integrity phenotype.

The other genes tested were endothelin-1, thrombomodulin-1 and PECAM1. None of these genes showed significant differences in mRNA expression in the KLF2-/-KLF4-/- or KLF2-/-KLF4+/- compared to WT or KLF2-/- embryos. Therefore, it is not likely that these genes are responsible for the diminished vascular integrity observed in KLF2-/-KLF4-/- and KLF2-/-KLF4+/- embryos.

## Discussion

At E9.5, KLF2-/-KLF4-/- embryos exhibit variable macroscopic and uniform microscopic hemorrhaging, and these are sometimes observed in KLF2-/-KLF4+/- embryos. KLF2-/-KLF4-/- and most KLF2-/-KLF4+/- embryos die by E10.5, which is earlier than either single knockout. Similarly, KLF1-/-KLF2-/- embryos also die sooner than either single knockout [24]. The KFL2-/-KLF4+/- phenotype is more variable and less severe than KLF2-/-KLF4-/-, indicating that the loss of an additional KLF4 allele negatively impacts mouse embryonic vascular development. Interestingly, KLF2+/-KLF4-/- embryos have a less severe phenotype than either KLF2-/-KLF4+/- or KLF2-/-KLF4-/- embryos, indicating that hemorrhaging at E9.5 occurs only with the complete loss of KLF2. A similar gene dosage effect is also evident in double mutant Hoxa-13 and Hoxd-13 mice [25]. Hoxa-13-/-Hoxd-13-/- and Hoxa-13-/-Hoxd-13+/- mutations are embryonic lethal. Hoxa-13+/-Hoxd-13-/- mice reach adulthood but have more severe abnormalities of the genitourinary and digestive systems than seen in Hoxa-13-/- or Hoxa-13+/-Hoxd-13+/- double heterozygote mice.

Interestingly, loss of blood vessel integrity is observed only in the primary head vein in E9.5 KLF2-/-KLF4-/- embryos. The significance of this observation is somewhat diminished by the fact that many vessels throughout the KLF2-/-KLF4-/- embryo exhibit gross hemorrhaging by E10.5, coincident with embryonic demise. The primary head vein is one of the major blood vessels at E9.5, but there is no evidence that it is subject to higher fluid shear force.

The most prominent effect on gene expression that we observed in KLF2-/-KLF4-/- compared to KLF2-/- and WT E9.5 embryos is a reduction in eNOS mRNA. eNOS regulates many processes relevant to vasculogenesis, including vasodilation, vascular smooth muscle tone, and endothelial homeostasis [26]. The roles of eNOS and nitric oxide in embryonic vascular development are less well-studied than in the adult. However, recently a role for VEGF-mediated regulation of eNOS in angioblast and embryonic endothelial cell proliferation has been discovered [27]. In E9.5 eNOS-GFP embryos, GFP can be detected in the vessels supplying the head, suggesting that eNOS could be expressed in primary head vein endothelial cells [28]. Blood vessels in embryos lacking KLF2 and KLF4 might not be able to withstand increasing fluid shear stress leading to their disruption, and this could be due in part to a reduction in eNOS. The lack of vascular integrity observed in KLF2-/-KLF4-/- embryos in this study is apparently not due simply to a lack of vascular smooth muscle or mural cell recruitment to vessels, because there is not yet a continuous layer of mesenchymal cells surrounding the primary head vein at E9.5. Although eNOS can be dispensable in early embryos, some eNOS-/- embryos die between E8.5 and E10.5, coincident with the time of death of KLF2-/-KLF4-/- embryos [29]. In the absence of KLF2 and KLF4 it is likely that a host of other endothelial regulators are down-regulated, in addition to eNOS, exacerbating the effect. Occludin and VEGFR2 mRNA are also reduced in E9.5 embryos lacking KLF2 and KLF4. Occludin is a tight junction protein, and its role in vascular integrity has been established [9]. Complete ablation of VEGFR2 leads to embryonic lethality and loss of vessel development [30]. Down-regulation of VEGFR2 in KLF2-/-KLF4-/- mice may further diminish eNOS expression, because VEGF signaling induces eNOS [27]. The data suggest that the loss of both KLF2 and KLF4 creates a situation where eNOS deficiency cannot be compensated, due to disruption of other endothelial regulators, resulting in loss of vascular integrity.

In cultured HUVEC cells, overexpression of KLF2 positively regulates the eNOS gene [31]. In our study in E9.5 mouse embryos, eNOS expression is regulated coordinately by KLF2 and KLF4, but is not diminished in the absence of KLF2 alone. Lee et al. also did not observe a reduction in eNOS mRNA in KLF2 endothelial conditional knockout mice at E11.5 [4]. Similarly, when the KLF2 gene is conditionally deleted in adult mice, occludin expression is reduced [32], but occludin mRNA is reduced in KLF2-/-KLF4-/- but not in KLF2-/- embryos, compared to WT. Evidently, KLF4 can partially compensate for KLF2 in regulating the eNOS and occludin genes in embryonic vascular endothelial cells, but not in the adult mouse or in tissue culture systems.

A limitation of this work is the use of knockout mice lacking expression of KLF2 and KLF4 in all cell types, rather than just endothelial cells. We cannot rule out the possibility that a lack of expression of KLF2 or KLF4 in cells other than endothelial cells may contribute to the vascular phenotype. For example, in adult mice, KLF2 and KLF4 are expressed in monocytes and macrophages [33-35]; it is not known if they are expressed in primitive phagocytes, and whether this could have a role in vasculogenesis. Furthermore, KLF4 is expressed in vascular smooth muscle cells [12], and could potentially be expressed and have a function in mesenchymal precursors to these cells at E9.5. However, only endothelial cells are specifically known to express both KLF2 and KLF4 at E9.5, so the most parsimonious explanation is that these genes interact in endothelial cells, resulting in the phenotype exhibited in double mutant embryos. In the future, a double conditional knockout mouse model could be used to determine if KLF2 and KLF4 have cell autonomous roles in the vascular endothelium.

## Conclusions

This study begins to define the roles of KLF2 and KLF4 in the embryonic development of blood vessels. The two genes interact to maintain an intact endothelial layer. KLF2 and KLF4 positively regulate the eNOS, VEGFR2 and occludin genes, and this may be required to establish or maintain vascular integrity. KLF2 and KLF4 could regulate these genes through direct or indirect mechanisms, and this is a question for future study.

## Methods

### Animal ethics

The animal experiments were approved by the Virginia Commonwealth University Institutional Animal Care and Use Committee, under protocol number AM10347.

### Generation of KLF2/KLF4 Mice

The KLF2 KO mouse model was developed by targeting the KLF2 gene with the hypoxanthine phosphoribosyl-transferase (Hprt) gene [6]. KLF4 KO mice were produced using a targeting vector containing a PGK promoter-driven neomycin gene to delete exons 2, 3, and a portion of exon 1 through homologous recombination in ES cells [11]. KLF2 and KLF4 are located on mouse chromosomes 8 and 4, respectively, and therefore segregate independently in meiosis. KLF2+/- and KLF4+/- mice were bred to generate KLF2+/-KLF4+/- double heterozygous mice, which were then bred to generate homozygous KLF2-/-KLF4-/- double knockout embryos. All embryos were in undefined mixed genetic background with approximately 50% FVB/N character.

Embryos were transferred to tooled neck glass vials or cryotubes and either quick-frozen in liquid nitrogen for analysis via quantitative reverse transcriptase polymerase chain reaction (qRT-PCR) or fixed for microscopy and electron microscopy. Tissue quick-frozen in liquid nitrogen was stored at -80°C until processing.

### Light and electron microscopy

Embryos were fixed in 2% PFA/0.5% glutaraldehyde and embedded in eponate 12 resin. Plastic-embedded specimens were cut in transverse section (cross-section) at 6 μm thickness on a Sorvall JB4 Microtome. The tissue sections were stained with a solution composed of 1% sodium borate, 1% azure II, 1% toluidine, and 1% methylene and photographed with an Olympus DP71 digital camera, mounted to an Olympus BX41 compound microscope, visualized with Olympus DP Controller 3.2.1.276 imaging software.

For transmission electron microcopy (TEM), thin sections were cut at 100 nm on a LKB 2128 Ultratome, and stained with 5% uranyl acetate and Reynold’s lead citrate. Images were made using a JEOL JEM-1230 TEM and Gatan Ultrascan 4000 digital camera at 1,000 – 8,000X.

### qRT-PCR

RNA was prepared from E9.5 whole embryos, and quantitative RT-PCR (qRT-PCR) was performed as previously described [7]. Primer sequences of the tested genes are as follows: eNOS 5′-CTGCCACCTGATCCTAACTTG-3′ and 5′-CAGCCAAACACCAAAGTCATG-3′; Endothelin 5′ CCTTAAGGGCCAGTTCAGGT 3′ and 5′ CTCTGCCAAGGATCGTGTTT 3′; Thrombomodulin-1 5′AAACACGATCCTTGGCAGAG 3′ and 5′ CCTAAGGGAGTCACGTGCAA 3′; VEGFR2 5′ AAACACGATCCTTGGCAGAG 3′ and 5′ GACTGGCCTAAGGGAGTCAC 3′; Occludin 5′-CACACTTGCTTGGGACAGAGG-3′ and 5′-TGAGCCGTACATAGATCCAGGAGC-3′; PECAM1 5′ TCCATGTCCCGAGAAGAGCAG 3′ and 5′ GCAGCGGGGTTTAAAATTG 3′ forward and reverse primers respectively. The normalized data were scaled by setting the WT average to a value of 100.

### Statistical analysis

The Student’s t-test was used for statistical analyses. Standard deviation was used to measure deviation from the mean, for all experiments. For all of the statistical tests, p values ≤ 0.05 were considered significant.

## Competing interests

The authors have no competing interests.

## Authors’ contributions

AC, BC, MK, SF, JH and JL participated in initial discovery and hypothesis development. AC, BC, JH and JL conceived the experiments. AC, BC and GE performed the light microscopy and histology experiments. BC and JH executed the electron microscopy experiments. AC carried out the gene expression assays. AC, BC, JH and JL analyzed the data. AC, BC, JH and JL wrote the manuscript. All authors read and approved the final manuscript.

## References

[B1] Rossant J Howard L Signaling pathways in vascular development Annu Rev Cell Dev Biol 2002 18 541 573 10.1146/annurev.cellbio.18.012502.105825 12142271

[B2] Cleaver O Melton DA Endothelial signaling during development Nat Med 2003 9 661 668 10.1038/nm0603-661 12778164

[B3] Drake CJ Fleming PA Vasculogenesis in the day 6.5 to 9.5 mouse embryo Blood 2000 95 1671 1679 10688823

[B4] Lee JS Yu Q Shin JT Sebzda E Bertozzi C Chen M Mericko P Stadtfeld M Zhou D Cheng L Graf T MacRae CA Lepore JJ Lo CW Kahn ML Klf2 is an essential regulator of vascular hemodynamic forces in vivo Dev Cell 2006 11 845 857 10.1016/j.devcel.2006.09.006 17141159

[B5] Kuo CT Veselits ML Barton KP Lu MM Clendenin C Leiden JM The LKLF transcription factor is required for normal tunica media formation and blood vessel stabilization during murine embryogenesis Genes Dev 1997 11 2996 3006 10.1101/gad.11.22.2996 9367982 PMC316695

[B6] Wani MA Means RTJ Lingrel JB Loss of LKLF function results in embryonic lethality in mice Transgenic Res 1998 7 229 238 10.1023/A:1008809809843 9859212

[B7] Chiplunkar AR Lung TK Alhashem Y Koppenhaver BA Salloum FN Kukreja RC Haar JL Lloyd JA Krüppel-like factor 2 is required for normal mouse cardiac development PLoS One 2013 8 e54891 10.1371/journal.pone.0054891 23457456 PMC3573061

[B8] Wu J Bohanan CS Neumann JC Lingrel JB KLF2 transcription factor modulates blood vessel maturation through smooth muscle cell migration J Biol Chem 2008 283 3942 3950 18063572 10.1074/jbc.M707882200

[B9] Lin Z Natesan V Shi H Dong F Kawanami D Mahabeleshwar GH Atkins GB Nayak L Cui Y Finigan JH Jain MK Krüppel-like factor 2 regulates endothelial barrier function Arterioscler Thromb Vasc Biol 2010 30 1952 1959 10.1161/ATVBAHA.110.211474 20651277 PMC3095948

[B10] Ehlermann J Pfisterer P Schorle H Dynamic expression of Krüppel-like factor 4 (Klf4), a target of transcription factor AP-2alpha during murine mid-embryogenesis Anat Rec A: Discov Mol Cell Evol Biol 2003 273 677 680 12845703 10.1002/ar.a.10089

[B11] Segre JA Bauer C Fuchs E Klf4 is a transcription factor required for establishing the barrier function of the skin Nat Genet JID - 9216904 1999 22 356 360 10.1038/11926 10431239

[B12] Liu Y Sinha S McDonald OG Shang Y Hoofnagle MH Owens GK Krüppel-like factor 4 abrogates myocardin-induced activation of smooth muscle gene expression J Biol Chem 2005 280 9719 9727 15623517 10.1074/jbc.M412862200

[B13] Yoshida T Kaestner KH Owens GK Conditional deletion of Krüppel-like factor 4 delays downregulation of smooth muscle cell differentiation markers but accelerates neointimal formation following vascular injury Circ Res 2008 20 102 1548 1557 18483411 10.1161/CIRCRESAHA.108.176974 PMC2633447

[B14] Kawai-Kowase K Owens GK Multiple repressor pathways contribute to phenotypic switching of vascular smooth muscle cells Am J Physiol Cell Physiol 2007 292 C59 C69 16956962 10.1152/ajpcell.00394.2006

[B15] Yoshida T Gan Q Franke AS Ho R Zhang J Chen YE Hayashi M Majesky MW Somlyo AV Owens GK Smooth and cardiac muscle-selective knock-out of Krüppel-like factor 4 causes postnatal death and growth retardation J Biol Chem 2010 285 21175 21184 10.1074/jbc.M110.112482 20439457 PMC2898332

[B16] Yoshida T Gan Q Owens GK Krüppel-like factor 4, Elk-1, and histone deacetylases cooperatively suppress smooth muscle cell differentiation markers in response to oxidized phospholipids Am J Physiol Cell Physiol 2008 295 C1175 C1182 10.1152/ajpcell.00288.2008 18768922 PMC2584997

[B17] Dekker RJ van Thienen JV Rohlena J de Jager SC Elderkamp YW Seppen J de Vries CJ Biessen EA van Berkel TJ Pannekoek H Horrevoets AJ Endothelial KLF2 links local arterial shear stress levels to the expression of vascular tone-regulating genes Am J Pathol 2005 167 609 618 10.1016/S0002-9440(10)63002-7 16049344 PMC1603569

[B18] Villarreal G Jr Zhang Y Larman HB Gracia-Sancho J Koo A Garcia-Cardena G Defining the regulation of KLF4 expression and its downstream transcriptional targets in vascular endothelial cells Biochem Biophys Res Commun 2010 391 984 989 10.1016/j.bbrc.2009.12.002 19968965 PMC4165389

[B19] Jiang J Chan YS Loh YH Cai J Tong GQ Lim CA Robson P Zhong S Ng HH A core Klf circuitry regulates self-renewal of embryonic stem cells Nat Cell Biol 2008 10 353 360 10.1038/ncb1698 18264089

[B20] McConnell BB Yang VW Mammalian Krüppel-like factors in health and diseases Physiol Rev 2010 90 1337 1381 10.1152/physrev.00058.2009 20959618 PMC2975554

[B21] Shesely EG Maeda N Kim HS Desai KM Krege JH Laubach VE Sherman PA Sessa WC Smithies O Elevated blood pressures in mice lacking endothelial nitric oxide synthase Proc Natl Acad Sci U S A 1996 93 13176 13181 10.1073/pnas.93.23.13176 8917564 PMC24066

[B22] Jin ZG Ueba H Tanimoto T Lungu AO Frame MD Berk BC Ligand-independent activation of vascular endothelial growth factor receptor 2 by fluid shear stress regulates activation of endothelial nitric oxide synthase Circ Res 2003 93 354 363 10.1161/01.RES.0000089257.94002.96 12893742

[B23] Feldman GJ Mullin JM Ryan MP Occludin: structure, function and regulation Adv Drug Deliv Rev 2005 57 883 917 10.1016/j.addr.2005.01.009 15820558

[B24] Basu P Lung TK Lemsaddek W Sargent TG Williams DC Jr Basu M Redmond LC Lingrel JB Haar JL Lloyd JA EKLF and KLF2 have compensatory roles in embryonic β-globin gene expression and primitive erythropoiesis Blood 2007 110 3417 3425 10.1182/blood-2006-11-057307 17675555 PMC2200909

[B25] Warot X Fromental-Ramain C Fraulob V Chambon P Dolle P Gene dosage-dependent effects of the Hoxa-13 and Hoxd-13 mutations on morphogenesis of the terminal parts of the digestive and urogenital tracts Development 1997 124 4781 4791 9428414 10.1242/dev.124.23.4781

[B26] Bautch VL VEGF-directed blood vessel patterning: from cells to organism Cold Spring Harb Perspect Med 2012 2 a006452 22951440 10.1101/cshperspect.a006452 PMC3426816

[B27] Gentile C Muise-Helmericks RC Drake CJ VEGF-mediated phosphorylation of eNOS regulates angioblast and embryonic endothelial cell proliferation Dev Biol 2013 373 163 175 10.1016/j.ydbio.2012.10.020 23103584 PMC3523730

[B28] Teichert AM Scott JA Robb GB Zhou YQ Zhu SN Lem M Keightley A Steer BM Schuh AC Adamson SL Cybulsky MI Marsden PA Endothelial nitric oxide synthase gene expression during murine embryogenesis: commencement of expression in the embryo occurs with the establishment of a unidirectional circulatory system Circ Res 2008 103 24 33 10.1161/CIRCRESAHA.107.168567 18556578

[B29] Jones EA The initiation of blood flow and flow induced events in early vascular development Semin Cell Dev Biol 2011 22 1028 1035 10.1016/j.semcdb.2011.09.020 22001248

[B30] Shalaby F Rossant J Yamaguchi TP Gertsenstein M Wu XF Breitman ML Schuh AC Failure of blood-island formation and vasculogenesis in Flk-1-deficient mice Nature 1995 376 62 66 10.1038/376062a0 7596435

[B31] Dekker RJ Boon RA Rondaij MG Kragt A Volger OL Elderkamp YW Meijers JC Voorberg J Pannekoek H Horrevoets AJ KLF2 provokes a gene expression pattern that establishes functional quiescent differentiation of the endothelium Blood 2006 107 4354 4363 10.1182/blood-2005-08-3465 16455954

[B32] Shi H Sheng B Zhang F Wu C Zhang R Zhu J Xu K Kuang Y Jameson SC Lin Z Wang Y Chen J Jain MK Atkins GB Krüppel-like factor 2 protects against ischemic stroke by regulating endothelial blood brain barrier function Am J Physiol Heart Circ Physiol 2013 304 H796 H805 10.1152/ajpheart.00712.2012 23335794 PMC3602774

[B33] Mizejewski GJ Biological roles of alpha-fetoprotein during pregnancy and perinatal development Exp Biol Med (Maywood) 2004 229 439 463 15169963 10.1177/153537020422900602

[B34] Feinberg MW Cao Z Wara AK Lebedeva MA SenBanerjee S Jain MK Krüppel-like factor 4 is a mediator of proinflammatory signaling in macrophages J Biol Chem 2005 280 38247 38258 10.1074/jbc.M509378200 16169848

[B35] Feinberg MW Wara AK Cao Z Lebedeva MA Rosenbauer F Iwasaki H Hirai H Katz JP Haspel RL Gray S Akashi K Segre J Kaestner KH Tenen DG Jain MK The Krüppel-like factor KLF4 is a critical regulator of monocyte differentiation EMBO J 2007 26 4138 4148 10.1038/sj.emboj.7601824 17762869 PMC2230668

